# Association of insulin resistance with the accumulation of saturated IMCL: a comparison with other fat stores

**DOI:** 10.1002/nbm.5117

**Published:** 2024-02-14

**Authors:** Mueed Azhar, Laura P.E. Watson, Emanuella De-Lucia Rolfe, Michele Ferraro, Katherine Carr, Jieniean Worsley, Chris Boesch, Leanne Hodson, Krishna K. Chatterjee, Graham J. Kemp, David B. Savage, Alison Sleigh

**Affiliations:** 1 Wolfson Brain Imaging Centre, Department of Clinical Neurosciences, University of Cambridge, Cambridge Biomedical Campus, Cambridge, UK; 2 National Institute for Health and Care Research Cambridge Clinical Research Facility, Cambridge University Hospitals NHS Foundation Trust, Cambridge Biomedical Campus, Cambridge, UK; 3 Metabolic Research Laboratories, Wellcome Trust-MRC Institute of Metabolic Science, University of Cambridge, Cambridge Biomedical Campus, Cambridge, UK; 4 MRC Epidemiology Unit, University of Cambridge, Cambridge Biomedical Campus, Cambridge, UK; 5 Departments of Clinical Research and Radiology AMSM, University Bern, Bern, Switzerland; 6 Oxford Centre for Diabetes, Endocrinology and Metabolism (OCDEM), NIHR Oxford Biomedical Research Centre, Churchill Hospital, University of Oxford, UK; 7 Department of Musculoskeletal & Ageing Science, University of Liverpool, Liverpool, UK

**Keywords:** Intramyocellular lipid, insulin resistance, saturation, ectopic, adipose tissue

## Abstract

It has been shown using ^1^H MRS that, in a group of females, whole-body insulin resistance was more closely related to accumulation of saturated intramyocellular lipid (IMCL) than to IMCL concentration alone. This has not been investigated in males. We investigated whether age- and BMI-matched healthy males differ from the previously-reported females in IMCL composition (measured as CH_2_:CH_3_) and IMCL concentration (measured as CH_3_), and in their associations with insulin resistance. We ask whether saturated IMCL accumulation is more strongly associated with insulin resistance than other ectopic and adipose tissue lipid pools, and remains a significant predictor when these other pools are taken into account. In this group of males, who had similar overall insulin sensitivity to the females, IMCL was similar between sexes. The males demonstrated similar and even stronger associations of IMCL with insulin resistance, supporting the idea that a marker reflecting the accumulation of saturated IMCL is more strongly associated with whole-body insulin resistance than IMCL concentration alone. However, this marker ceased to be a significant predictor of whole-body insulin resistance after consideration of other lipid pools, which implies that this measure carries no more information in practice than the other predictors we found, such as intrahepatic lipid and visceral adipose tissue. As the marker of saturated IMCL accumulation appears to be related to these two predictors and has a much smaller dynamic range, this finding does not rule out a role for it in the pathogenesis of insulin resistance.

## 1 Introduction

Since the discovery over two decades ago that proton magnetic resonance spectroscopy (^1^H-MRS) can non-invasively distinguish extra- from intra-myocellular lipids (IMCL)^1,2^, there have been many studies of the physiology and pathophysiology associated with IMCL. Of particular interest were early reports^3–5^ of associations between soleus IMCL and insulin resistance independent of fat mass, especially as skeletal muscle is the main site for postprandial glucose disposal^6^. Interest declined when other studies reported that this association was not always robust^7,8^, and it was discovered that the IMCL pool size can be modulated by multiple factors including diet and exercise^9^. These studies used the fatty acid methylene (CH_2_) resonance as a proxy to estimate the concentration of IMCL (due to its higher signal intensity than other proton resonances in triglyceride); however, estimates of the IMCL pool size made using this method are influenced by its fatty acid composition, leading to uncertainty over the interpretation of the reported associations, or their lack, with insulin sensitivity.

Recently we described and validated a ^1^H MRS method that, with appropriate prior knowledge applied to good quality spectra acquired at 3T with a short echo time, can provide measures of both IMCL composition and concentration independent of composition^10^. This compositional measure of IMCL compares the CH_2_ resonance at 1.3 ppm (which reflects both concentration and composition) with the CH_3_ resonance at 0.9 ppm (which is independent of composition), and is influenced mainly by the saturation of the fatty acid chains and to a lesser extent their chain length^10^. Using this method in female participants with insulin resistance due to lipodystrophy as well as female healthy controls, we showed that the accumulation of saturated IMCL is more closely related with whole-body insulin resistance than IMCL concentration alone^11^. Importantly, this association persisted within the female control group alone. This relationship has yet to be studied in males.

The association of obesity and insulin resistance is firmly established, and numerous fat depots including visceral adipose tissue (‘central obesity’) and intrahepatocellular lipid (IHL) have been found to be strong predictors of metabolic syndrome ^12,13^. However, few studies have attempted to tease out which of the many fat pools are the best predictors of insulin resistance ^14,15^.

In this study, we aim to determine whether age- and BMI- matched healthy males differ from females in IMCL composition and concentration independent of composition, and whether they show similar associations with insulin resistance. We also investigate whether the marker of saturated IMCL accumulation is more strongly associated with insulin resistance than other ectopic and adipose tissue pools, and whether it remains a significant predictor when these factors are taken into account.

## 2 Materials and Methods

### 2.1 Participants

30 male individuals, age- and BMI- matched at a group level to 41 females, were recruited through advertisement. Data for IMCL measures from these 41 females have been published in a previous report ^11^. Current smoking, drug or alcohol addiction, any current or past medical disorder or medication that could influence measurements, and standard MRI contraindications were exclusion criteria. The East of England Cambridge Central Ethics Committee approved the healthy volunteer reference measurement studies (06/Q0108/84). The studies were conducted following the guidelines of the Declaration of Helsinki, and all participants gave written informed consent.

### 2.2 Protocol

Volunteers maintained normal diet for 3 days and refrained from alcohol and vigorous activity for at least 19 hours before attending the National Institute for Health and Care Research (NIHR) Cambridge Clinical Research Facility. In the morning (between 0800 and 0900*)* fasting blood samples were taken and a light breakfast of toast or cereal served prior to ^1^H MRS.

Dual-energy X-ray absorptiometry evaluated body composition, using Lunar Prodigy enCORE v.12.5 (GE Healthcare, Madison, WI) and a Siemens 3T Verio scanner (Erlangen, Germany) was used for ^1^H MRS.

### 2.3 Intramyocellular lipid (IMCL)

Point-REsolved Spectroscopy (PRESS) used a short echo time of 35 ms to obtain a water-suppressed (VAPOR) ^1^H spectrum (5 s repetition time to ensure no T_1_ weighting, 64 averages) from a 1.3 × 1.3 × 1.3 cm^3^ voxel, positioned avoiding visible fat on T1-weighted images in tibialis anterior (TA) and soleus (SOL) muscles. A combination of automated and manual shimming was performed, and data were collected using the standard PA coil. All spectra were free from eddy current effects and no eddy current correction was applied. The data were analysed in jMRUI ^16,17^, using the AMARES algorithm^18^ with identical prior-knowledge parameters. Starting values for tCr, EMCL CH_2_, IMCL CH_2_ frequencies were (3.02, 1.5, 1.29 ppm). Gaussian line shapes (except for water which used Lorentzian) were used, and soft constraints were applied on EMCL/IMCL CH_2_ frequencies and linewidths (0-30 Hz). CH_3_ frequencies and linewidths were based on prior knowledge relative to the CH_2_ resonance^19^, and all amplitudes were estimated. All peaks had relative phase set to zero. Clarification of these fitting parameters are outlined within Fitting Routine 1 of Supplementary Table 1 within^11^. On the Overall Phases card, phasing parameters PH0 and PH1 were both fixed to zero and weighting was applied to points 1-5 with no truncation. It was important that calibration of the frequency axis was such that the starting value dot was directly on the top of the tCr peak, thereby forming consistent starting frequency positions for the IMCL and EMCL resonances. The concentrations of IMCL CH_2_ and CH_3_ were determined by comparison with the CH_3_ of total creatine (TCr = creatine + phosphocreatine), which has chemical shift 3.0 ppm. As this resonance has different lineshapes in TA and SOL, quantification between muscles using a nominal [TCr] is not valid; instead, a TCr/water scaling factor was used for each muscle, established from a subset of participants who had non-water suppressed data sets^11^.

**Figure 1:**
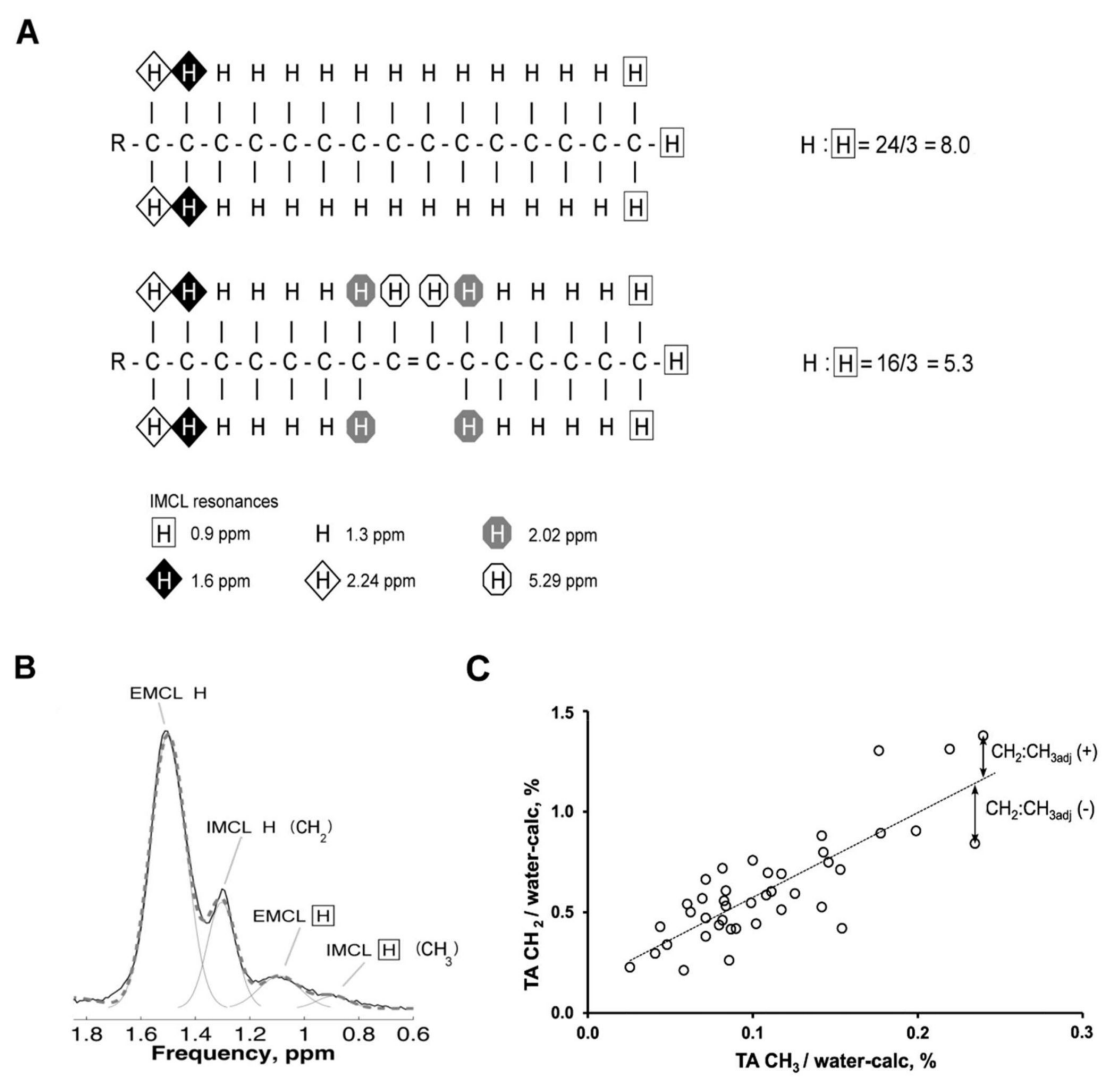
The ratio of CH_2_:CH_3_ is influenced primarily by the saturation of the fatty acids in triglyceride (TG). (A) The palmitoleic acid component of TG has a theoretical ratio of CH_2_ (at 1.3 ppm) to CH_3_ (at 0.9 ppm) of 16/3 = 5.3, lower than the equivalent ratio of palmitic acid = 8.0. The CH_2_:CH_3_ ratio is also influenced by chain length, but to smaller effect^10^. (B) Example soleus spectra and fit where EMCL is high (CH_2_/water: IMCL=2.3%, EMCL=7.7%). (C) Plot of tibialis anterior CH_2_ vs CH_3_ in female individuals. As the CH_2_:CH_3_ ratio varies with IMCL quantity, the CH_2_:CH_3_ marker is adjusted for quantity (CH_2_:CH_3adj_), which is defined as the vertical (CH_2_/water) distance from the regression line through the female data points (dotted line), and mainly reflects the accumulation of saturated IMCL^11^. Part of this figure has been reproduced from Thankamony et al.^10^, which is licensed under a Creative Commons Attribution 4.0 International License.

The CH_3_ IMCL resonance reflects IMCL concentration that is independent of composition, whereas the CH_2_ IMCL resonance reflects both concentration and composition. The ratio of the two (CH_2_:CH_3_), which we refer to as the ‘saturation index’, reflects the average composition of the IMCL pool. It was previously shown^11^ that the saturation index varies with IMCL concentration in female controls, and that this was independent of insulin sensitivity. In order to provide a more pathophysiologically meaningful measure, the ‘saturation index adjusted for quantity’ (CH_2_:CH_3adj_) was calculated as CH_2_ – (mCH_3_ + c), where m and c are the gradient and intercept, respectively, of the regression line through CH_2_ *vs* CH_3_ in the control data points. This can be visualised as the vertical deviation (CH_2_) from the regression line through the female CH_2_ *vs* CH_3_ points, where anything above the line is positive, below negative, and represents a measure of the accumulation of saturated IMCL (Figure 1). It was this measure that is most closely related to insulin resistance in healthy female individuals^11^.

### 2.4 Intrahepatic lipid (IHL)

The measurement of IHL was conducted using respiratory gated ^1^H MRS, using the CH_2_ peak at 1.3 ppm in relation to water, as previously described ^20^. In summary, PRESS voxel with cube length 1.5 cm, located within right posterior lobe, was used with echo-time 35 ms and repetition time 7 s such to enable participants to comfortably follow breathing instructions such that they were at hold at the end of expiration during the localisation and subsequent acquisition. Shimming was performed using Siemen’s GRE abdomen protocol, and data was collected using the standard Body Matrix coil.

### 2.5 Adipose tissue

Magnetic resonance imaging was used to measure abdominal subcutaneous (SCAT_abd_) and visceral (VAT) adipose tissue areas on a water-suppressed T1-weighted transaxial image located at the L4 vertebral level. Leg subcutaneous adipose tissue (SCAT_leg_) and intermuscular fat (IMF) were assessed from a single T1-weighted transaxial slice at the same axial position as the IMCL measurements. Areas of SCAT, VAT and IMF were calculated by a semi-automated method, using a threshold map in conjunction with manual input to distinguish between compartments using the software Analyze (AnalyzeDirect, Overland Park, KS).

### 2.6 Biochemistry

Fasting glucose and insulin concentrations were measured by standard clinical laboratory methods. The HOMA-IR was calculated as the product of fasting insulin (mU/l) and fasting glucose (mmol/l), divided by 22.5.

### 2.7 Statistics

Statistical analysis was performed in SPSS Statistics 28 (IBM, Armonk, NY), setting significance at P < 0.05. Data not normally distributed (by Shapiro-Wilk test) were logarithmically transformed prior to statistical analysis. Two-tailed independent samples t-tests were used to test for sex differences. Pearson correlation coefficient were used to measure associations, and P adjusted for multiple comparisons by the Benjamini-Hochberg^21^ method. Significant predictors were assessed by stepwise linear regression, starting with the measure that had the strongest association.

## 3 Results

### 3.1 Participants

The age- and BMI-matched male participants had similar whole-body insulin sensitivity measures to female participants: HOMA-IR (1.14±0.13 vs 1.1±0.17, Table 1). As expected, males had higher total and fat-free mass, while females had a higher fat mass. Serum triglyceride concentration was similar in females and males, but HDL-cholesterol concentration was lower in males (Table 1).

### 3.2 Effect of sex on lipid measures

**Figure 2:**
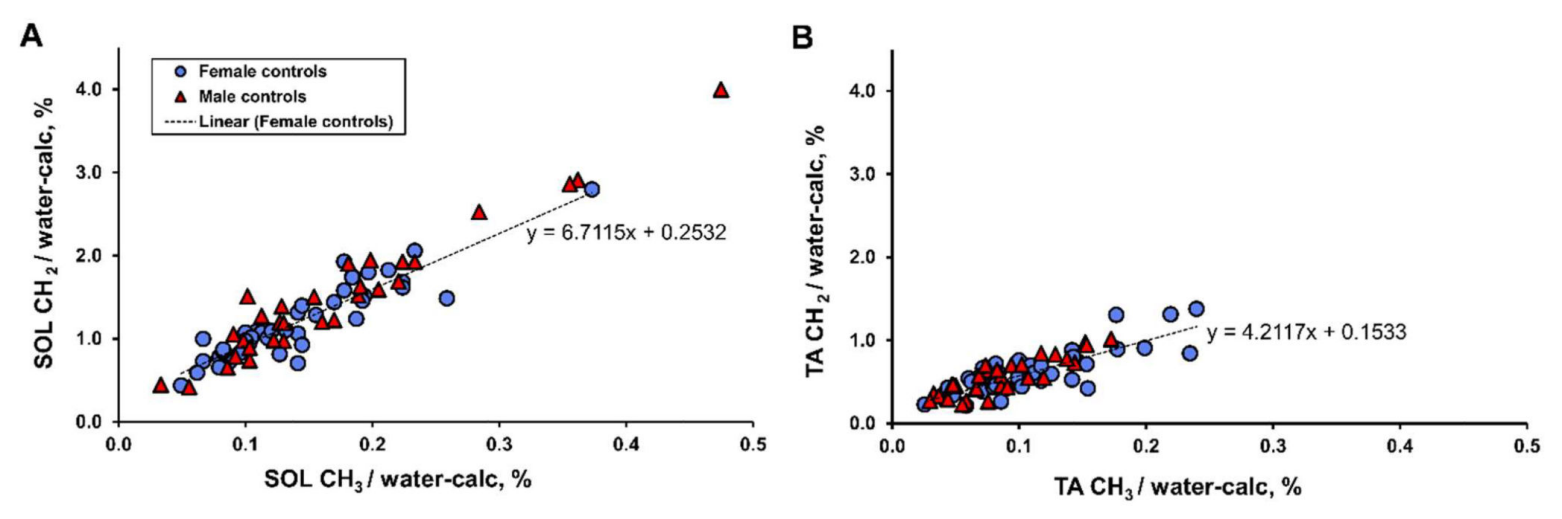
Soleus (A) and tibialis anterior (B) IMCL CH_2_ (at 1.3 ppm) and CH_3_ (at 0.9 ppm) in both male and female controls. Dotted line = linear regression of female control data points.

Table 1 gives female and male lipid measures. There were no sex differences in the linewidth of tCr, which when combined had a (mean ± SEM) linewidth of (10.2 ± 0.3) Hz in the soleus, and (7.9 ± 0.3) Hz in the tibialis anterior. Figure 2 shows the relationship of IMCL CH_2_ *vs* CH_3_, which can be thought of as a graphical demonstration of IMCL composition.

There were no statistically significant sex differences in IMCL measures, but males tended to have higher soleus IMCL CH_2_:CH_3adj_ (P = 0.059), lower TA IMCL concentration (P = 0.055) and higher IMCL CH_2_:CH_3_ (P = 0.051) (Table 1, Figure 2). EMCL was significantly lower in TA of males.

There were no significant sex differences in intrahepatic lipid, intramuscular fat, or abdominal visceral fat, however, males had significantly less leg subcutaneous fat and a tendency for less abdominal subcutaneous fat (P = 0.066, Table 1).

### 3.3 Relation of lipid measure with insulin sensitivity

Table 2 shows the relationship of HOMA-IR and lipid measures in females and males separately, and together. Males alone, and males together with females, showed similar and stronger associations of IMCL with HOMA-IR compared with female controls. HOMA-IR was not associated with EMCL (CH_2_, CH_3_, or CH_2_:CH_3_) in either SOL or TA muscle (not presented). HOMA-IR was also not associated with VAT:SCAT ratio in either sex or combined (not presented).

Total fat mass, intrahepatic lipid, and leg subcutaneous fat had significant correlations with HOMA-IR in females, males, and together (Table 2). Other significant correlations included abdominal visceral fat (females, females and males together), and abdominal subcutaneous fat (female and male together).

Performing stepwise linear regression and starting with the measure that has the strongest association, the only significant predictors of HOMA-IR were VAT in females (R = 0.517, P < 0.001), IHL in males (R = 0.539, P = 0.004), and IHL and fat mass in females and males combined (R = 0.513, IHL P = 0.010, fat mass P = 0.039).

**Figure 3:**
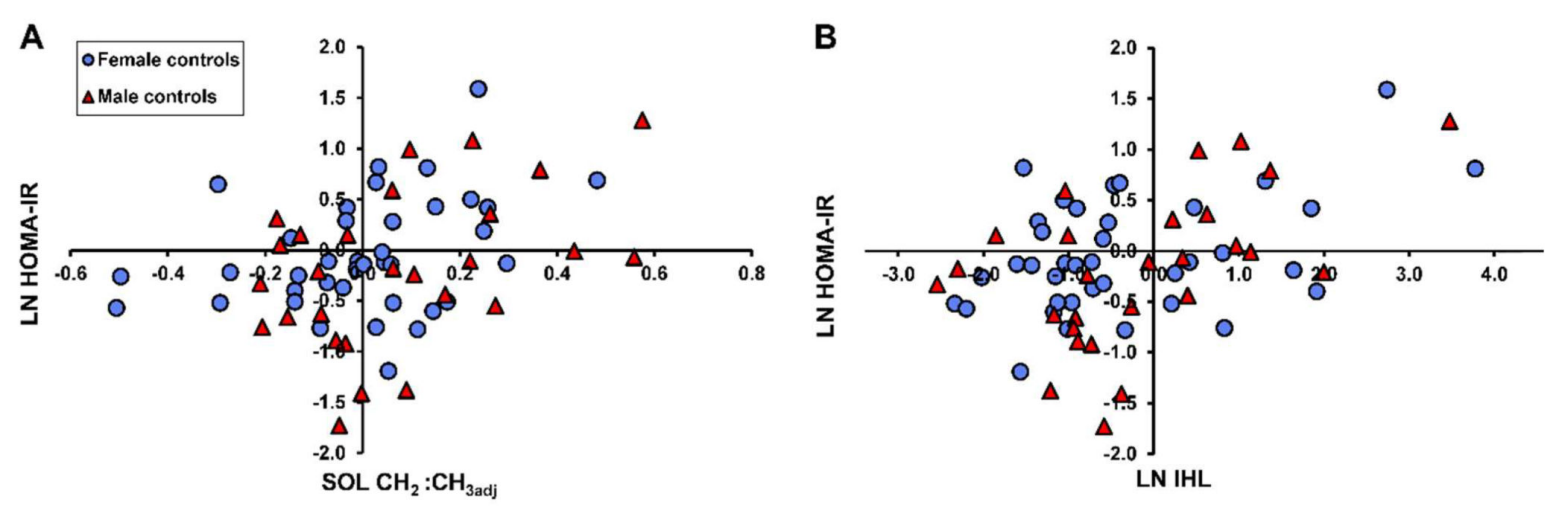
Relation of (A) soleus compositional adjusted saturation index (CH_2_:CH_3adj_) and (B) intrahepatic lipid with HOMA-IR in female (blue) and male controls (red). HOMA-IR correlation coefficients are shown in Table 2.

Figure 3A shows the correlation of HOMA-IR to soleus compositional adjusted saturation index (CH_2_:CH_3adj_) in females (R = 0.342, uncorrected P = 0.036), males (R = 0.429, uncorrected P = 0.026), and females and males together (R = 0.358, uncorrected P = 0.003). Figure 3 B shows the correlation of HOMA-IR to intrahepatic lipid, in females (R = 0.429, uncorrected P = 0.007), males (R = 0.511, uncorrected P = 0.006), and females and males together (R = 0.452, uncorrected P <0.001).

### 3.4 Inter-relation of IMCL with different lipid measures

Tables 3 and 4 show the associations between IMCL concentration, and adjusted IMCL composition, respectively, with other lipid measures. IMCL concentration tends to be associated with adipose tissue stores. Soleus IMCL concentration was significantly correlated with intramuscular fat and total fat mass (females, males, and females and males together), abdominal subcutaneous fat (females, female and males together), visceral fat (males, females and males together) and leg subcutaneous fat (males). Whereas TA IMCL concentration only showed significant associations with intramuscular fat, leg subcutaneous fat, and total fat mass (females and males together).

By contrast, the IMCL composition adjusted for quantity, females demonstrated a tendency with VAT_abd_ (SOL) and both IHL and VAT_abd_ (TA). In males, IMCL composition adjusted for quantity showed a strong association with IHL and tendency with VAT_abd_, SCAT_abd_, DXA FM, and TG (SOL); yet there was no correlation with other body fat depots (TA). Across both sexes and muscles, there was a significant correlation between adjusted IMCL composition and both IHL and visceral adipose tissue.

Performing stepwise linear regression revealed that the only significant predictors of SOL IMCL composition adjusted for quantity, were VAT in females (R = 0.340, P = 0.030), IHL in males (R = 0.556, P = 0.001), and IHL in males and females combined (R = 0.422, P < 0.001), with a tendency for TG in males and females combined (R = 0.467, IHL P = 0.011, TG P = 0.092). Stepwise linear regression revealed that the only significant predictors of TA IMCL composition adjusted for quantity were IHL in females (R = 0.394, P = 0.011), and IHL in females and males combined (R = 0.363 P = 0.002).

## 4 Discussion

Using a ^1^H MRS marker that we have previously validated at short echo-time (where the CH_2_:CH_3_ ratio has greater dynamic range than at longer echo-times), we investigated the effect of sex on IMCL composition and concentration (independent of composition) by comparing 30 age- and BMI-matched males with 41 females, asking whether the associations of saturated IMCL with whole-body insulin resistance previously reported in females also hold true in the males. With this group of males, who had similar insulin sensitivity to the females, the IMCL measures were similar between sexes. There was a tendency (P < 0.1) for males to have lower IMCL concentration (CH_3_) and higher saturation (CH_2_:CH_3_) in the tibialis anterior, and higher composition adjusted for quantity (CH_2_:CH_3adj_) (i.e. more saturated lipid) in soleus. Although these measures have not been reported in males, a study that used the CH_2_ resonance (which reflects a combination of concentration and composition) is in partial agreement with our findings, demonstrating similar IMCL in tibialis anterior^22^, and higher IMCL in soleus^22^, of age- and BMI-matched males *vs* females.

Compared with our previously-reported females, the males we studied demonstrated similar, and stronger, relations to whole-body insulin resistance; in addition to the soleus composition adjusted for quantity (CH_2_:CH_3adj_), the tibialis anterior CH_2_:CH_3adj_ and concentration independent of composition (CH_3_) also correlated with insulin resistance. Other body fat markers that significantly associated with whole-body insulin resistance included intrahepatic lipid, total fat mass and leg subcutaneous fat in males and females separately and combined, as well as visceral adipose tissue (females & combined) and abdominal adipose tissue (combined only).

The strong association of intrahepatic lipid with insulin resistance is well-known^23,24^; however, the association with leg subcutaneous adipose tissue may seem surprising as storage of triglycerides in subcutaneous adipose tissue is thought to be protective, particularly in the legs^25,26^. However, there are other reports that this pool correlates with insulin resistance ^27^. It is conceivable that, although beneficial to store lipid in leg subcutaneous adipose tissue, that overall obesity dominates this finding; this is supported by the finding that this association disappears when the leg subcutaneous fat is expressed relative to total fat mass (all p > 0.4). Visceral adipose tissue was most strongly associated with insulin resistance in females, and was significantly associated in females and males combined, but did not reach significance in males. This is consistent with a previous report that although the female visceral adipose pool is generally smaller than in males, the expansion of this lipid pool in females poses a greater metabolic risk^28^. It is notable that other than soleus IMCL concentration, intramuscular lipid was the only other lipid pool not significantly associated with insulin resistance. Previous reports ^29^ have shown correlations of this pool with both insulin resistance and visceral adipose tissue; however, as these individuals were generally older with a larger BMI than our cohort, it is possible that accumulation of this pool is secondary to visceral fat expansion or occurs later in life.

Attempting to disentangle the relationships with insulin resistance by stepwise regression revealed that the only significant predictors were visceral adipose tissue in females, intrahepatic lipid in males, and intrahepatic lipid and fat mass in females and males combined. This is in agreement with previous findings which show intrahepatic lipid and visceral adipose tissue^14,15^, as well as body fat and IMCL^15^ to be independent predictors of insulin resistance in males and females. In both these studies^14,15^, intrahepatic lipid was the most significant predictor of insulin resistance.

We did not find the adjusted IMCL composition marker or IMCL concentration to be significant predictors of insulin resistance, which implies these measures carry no more information in practice than the predictors we found. However, the performance of any marker depends on factors such as measurement precision and accuracy, biological variation and dynamic range in addition to the underlying biology; and significant dependent variables with larger dynamic ranges and/or precision have the potential to mask other predictors. Investigating this possibility revealed that the adjusted IMCL compositional marker was indeed associated with intrahepatic lipid and visceral adipose tissue, and that intrahepatic lipid was its only predictor, implying a close relationship between the two depots.

Although a link between ectopic lipid accumulation and insulin resistance is accepted^30,31^, the favoured hypothesis is that, rather than triglyceride itself, lipid intermediates such as diacylglycerol (DAG) and ceramide are suggested to be involved in insulin resistance^32^. Saturated fat has been implicated in the pathogenesis of metabolic disease^33,34^, however, the mechanism by which this acts (e.g. inflammation, increases in ceramide, or increases in DAG due to DNL), or is accumulated (e.g. diet, DNL), is unclear. However, it is important to recognise that although our CH_2_:CH_3_ marker is primarily influenced by degree of saturation, it is also influenced by increasing chain length (the opposite to CH_3_:CH_2_ which represents degree of unsaturation and shorter chain length)^10^. This matches the profile of both preferred fatty acid mobilisation and oxidation^35,36^ and may therefore, independent of quantities of saturated fat, be physiologically relevant. Indeed, although we have validated our IMCL CH_3_:CH_2_ marker against theoretical CH_3_:CH_2_ values in IMCL/EMCL simulated phantoms^10^, this does not necessarily represent quantities of absolute saturated fat in oils that do not contain physiologically plausible SFA/MUFA/PUFA percentages.

We chose to study associations in a healthy population in an attempt to look at early markers associated with insulin resistance; however, there was still a wide range of BMI including both normal and overweight individuals, and it is possible that studies that capture information at even earlier points in the process, or that can probe these relationships, may be needed to provide more meaningful mechanistic information. Studies of lean offspring of type 2 diabetics^3,4^ as well as studies in prepubertal children^37,38^ have indeed shown that soleus IMCL CH_2_, a marker of both composition and content, is increased and/or associated with insulin resistance, supporting the idea that the accumulation of saturated IMCL could be mechanistically involved very early on. In fact, in one study in prepubertal children it has been suggested that familial factors have more influence on IMCL than the child’s current anthropometry^38^, giving rise to an idea that children develop or inherit muscle metabolic characteristics that are associated with insulin resistance. This is supported by the observation study in infants, which demonstrated that maternal triglyceride levels were inversely associated with the infant’s muscle membrane unsaturation index^39^, suggesting that some skeletal muscle alterations may precede insulin resistance.

Our study has limitations. We did not acquire water-suppressed data in liver, so could not accurately measure the CH_3_ of IHL, and as such we used the standard CH_2_ peak. Also, although the adjusted IMCL compositional marker is primarily related to the saturation of the IMCL pool, no information is gathered regarding the ratios of saturated, monounsaturated, or polyunsaturated pools. In addition, although we have established which lipid pools most closely relate to insulin resistance, this cross-sectional study in healthy individuals does not provide any information on causality. We also acknowledge that the number of participants is small compared to epidemiological studies but is of similar size to other MR studies^14^.

Our discovery that males exhibit similar relationships of IMCL to insulin resistance as our previously-reported females that, supports the idea that the accumulation of saturated IMCL is more strongly associated with whole-body insulin resistance than IMCL concentration alone. Attempting to disentangle relationships of multiple other body fat depots, did not reveal the accumulation of saturated IMCL to be a significant predictor of whole-body insulin resistance, which implies this measure carries no more information in practice than the predictors we found, such as intrahepatic lipid and visceral adipose tissue. However, as the marker of accumulation of saturated IMCL appears to be related to these two predictors and has a much smaller dynamic range, this finding does not rule out saturated IMCL accumulation as part of the pathogenesis of insulin resistance.

## Figures and Tables

**Table 1:** Participant characteristics and lipid measurements

	Female participants n = 41	Male participants n = 30	P value
Age, y	35.5±2.0	34.7±2.1	0.823
BMI, kg/m^2^	24.4±0.6	24.8±0.6	0.518
Mass, kg	65.2±2.3	78.6±2.2	**<0.001**
Fat mass, kg	23.1±1.6	17.5±1.7	**0.009**
FFM, kg	42.1±0.9	61.0±1.3	**<0.001**
Triglyceride, mmol/l	0.92±0.07 ^a^	1.14 ± 0.15 ^b^	0.233
HDL-cholesterol, mmol/l	1.64±0.06 ^a^	1.39 ± 0.06 ^b^	**0.004**
Fasting glucose, mmol/l	4.56±0.06 ^a^	4.61±0.06 ^c^	0.523
Fasting insulin, pmol/l ^d^	38.6±4.2 ^a^	36.9±5.7 ^c^	0.358
HOMA-IR	1.14±0.13 ^a^	1.10±0.17 ^c^	0.416
**IMCL measures**			
***Soleus***			
Concentration (CH_3_/water), %	0.14±0.01	0.17±0.01	0.342
Concentration and composition (CH_2_/water), %	1.22±0.07	1.49±0.14	0.181
Composition (CH_2_:CH_3_)	8.80±0.26	9.09±0.34	0.461
Composition adjusted for quantity (CH_2_:CH_3adj_)	0.00±0.03	0.10±0.04	0.059
***Tibialis anterior***			
Concentration (CH_3_/water), %	0.11±0.01	0.09±0.01	0.055
Concentration and composition (CH_2_/water), %	0.61±0.04	0.56±0.04	0.406
Composition (CH_2_:CH_3_)	6.01±0.28	6.88±0.35	0.051
Composition adjusted for quantity (CH_2_:CH3adj)	0.00±0.02	0.04±0.02	0.203
**EMCL measures**			
Soleus CH_2_/water, %	2.22±0.19	2.40±0.23	0.556
Tibialis anterior CH_2_/water, %	2.30±0.19	1.30±0.14	**<0.001**
**Other lipid measures**			
Intrahepatic lipid (CH_2_/water), %	2.5±1.1	2.5±1.1	0.790
Intramuscular fat, cm^2^	2.2±0.2	2.6±0.4	0.735
Leg subcutaneous fat, cm^2^	29.9±1.9	15.1±1.1	**<0.001**
Abdominal visceral fat, cm^2^	38.6±3.7	53.0±6.8	0.115
Abdominal subcutaneous fat, cm^2^	207±18	170±19	0.066

Non-normally distributed variables were log-transformed before independent-samples t-test. Bold P values are statistically significant. Data are mean ± SEM. FFM, fat free mass; HOMA-IR, Homeostasis Model Assessment of Insulin Resistance; IMCL, intramyocellular lipid; EMCL, extramyocellular lipid; CH_3_ and CH_2_, methyl and methylene protons resonating at 0.9 ppm and 1.3 ppm, quantified as % of uncorrected water resonance.

a n = 38,

b n = 29,

c n = 27,

d To convert to mU/l divide by 6.945.

**Table 2:** Correlation coefficients of whole-body insulin resistance with lipid measures

		HOMA-IR	
	Females	Males	Females and males combined
	n = 38	n = 27	n = 65
**IMCL Concentration**			
Soleus (CH_3_)	0.080	0.054	0.057
Tibialis Anterior (CH_3_)	0.275	0.387 ‡	0.315 *
**IMCL Composition adjusted for quantity**			
Soleus (CH_2_:CH_3adj_)	0.342‡^a^	0.429 *	0.358 *
Tibialis Anterior (CH_2_:CH_3adj_)	0.260	0.447 *	0.306 *
**Other lipid measures**			
Intrahepatic lipid (CH_2_)	0.429 *	0.511 *	0.452 *
Intramuscular lipid	0.216	0.118	0.115
Leg subcutaneous fat	0.355 ‡	0.431 *	0.362 *
Abdominal visceral fat	0.517 *	0.292	0.367 *
Abdominal subcutaneous fat	0.210	0.329	0.293 ‡
DXA total fat mass	0.403 *	0.463 *	0.394 *

Adjusted P values:

‡ p<0.10,

* p<0.05

Associations were assessed by Pearson correlation coefficient and p-values adjusted for multiple comparisons by the Benjamini-Hochberg method. HOMA-IR, Homeostasis Model Assessment of Insulin Resistance; F, female; M, male; IMCL, intramyocellular lipid; CH_3_, methyl protons resonating at 0.9 ppm; CH_2_:CH_3adj_, CH_2_:CH_3_ saturation index adjusted for lipid quantity; DXA, dual-energy X-ray absorptiometry.

a Difference in statistical significance to ^11^ due to correction for multiple comparisons.

**Table 3:** Correlation coefficients of IMCL concentration with other lipid measures.

IMCL concentration (CH_3_)	Concentration (CH_3_)		Composition (CH_2_:CH_3adj_)		Hepatic lipid	Adipose tissue				Total FM	Plasma
	SOL	TA	SOL	TA	IHL	IMF	SCAT_leg_	VAT_abd_	SCAT_abd_	DXA FM	TG ^a^
**Females**											
SOL	-	0.427*	0.013	0.062	0.128	0.392*	0.301	0.237	0.399*	0.404*	-0.039
TA	0.427*	-	0.100	-0.020	0.197	0.336	0.323	0.117	0.179	0.261	0.017
**Males**											
SOL	-	0.184	0.455*	0.389	0.272	0.635*	0.452*	0.635*	0.360	0.367	0.127
TA	0.184	-	0.229	0.251	0.359	0.336	0.250	0.261	0.255	0.343	0.323
**Females and males**											
SOL	-	0.239	0.255	0.202	0.204	0.525*	0.200	0.466*	0.342*	0.367*	0.063
TA	0.239	-	0.074	0.024	0.231	0.294*	0.355*	0.111	0.237	0.310*	0.109

a n= 38F, 29M

Associations were assessed by Pearson correlation coefficient and p-values adjusted for multiple comparisons by the Benjamini-Hochberg method. Adjusted p values: p<0.10 in bold,

* p<0.05

CH_3_, methyl groups resonating at 0.9 ppm; CH_2_:CH_3adj_, compositional marker adjusted for quantity; SOL, soleus; TA, tibialis anterior; IHL, intrahepatic lipid; IMF, intramuscular lipid; SCAT_leg_, calf subcutaneous fat; VAT_abd_, abdominal visceral adipose tissue; SCAT_abd_, abdominal subcutaneous adipose tissue; FM, fat mass; DXA, dual-energy X-ray absorptiometry; TG, triglyceride.

**Table 4:** Correlation coefficients of composition adjusted for concentration with other lipid measures.

IMCL composition (CH_2_:CH_3adj_)	Concentration (CH_3_)		Composition (CH_2_:CH_3adj_)		Hepatic lipid	Adipose tissue				Total FM	Plasma
	SOL	TA	SOL	TA	IHL	IMF	SCAT_leg_	VAT_abd_	SCAT_abd_	DXA FM	TG ^a^
**Females**											
SOL	0.013	0.100	-	0.304	0.300	0.233	0.113	**0.340**	0.191	0.180	0.256
TA	0.062	-0.20	0.304	-	**0.394**	-0.120	0.210	**0.346**	0.192	0.181	0.119
**Males**											
SOL	**0.455**	0.229	-	0.557*	0.556*	0.266	0.360	**0.412**	**0.447**	**0.463**	**0.442**
TA	**0.389**	0.251	0.557*	-	0.288	0.221	0.141	0.188	0.141	0.223	0.206
**Females and males**											
SOL	**0.255**	0.074	-	0.411*	0.422*	**0.252**	0.015	0.402*	**0.254**	0.210	0.367*
TA	0.202	0.024	0.411*	-	0.363*	0.086	0.033	**0.290**	0.127	0.104	0.175

a n= 38F, 29M

Associations were assessed by Pearson correlation coefficient and p-values adjusted for multiple comparisons by the Benjamini-Hochberg method. Adjusted p values: p<0.10 in bold,

* p<0.05.

CH_3_, methyl groups resonating at 0.9 ppm; CH_2_:CH_3adj_, compositional marker adjusted for quantity; SOL, soleus; TA, tibialis anterior; IHL, intrahepatic lipid; IMF, intramuscular lipid; SCAT_leg_, calf subcutaneous fat; VAT_abd_, abdominal visceral adipose tissue; SCAT_abd_, abdominal subcutaneous adipose tissue; FM, fat mass; DXA, dual-energy X-ray absorptiometry; TG, triglyceride.

## Data Availability

The data that support the findings of this study are available from the corresponding author upon reasonable request.

## Abbreviations

DXA FM: dual-energy X-ray absorptiometry fat mass
HOMA-IR: Homeostatic Model Assessment for Insulin Resistance
IHL: intrahepatic lipid
IMCL: intramyocellular lipid
IMF: intramuscular fat
SCAT: subcutaneous adipose tissue
SOL: soleus
TA: tibialis anterior
TCr: total creatine
TG: triglyceride
VAT: visceral adipose tissue
